# Oxidative Stress in Rice (*Oryza sativa*): Mechanisms, Impact, and Adaptive Strategies

**DOI:** 10.3390/plants14101463

**Published:** 2025-05-14

**Authors:** Lady Edlenill J. Tavu, Mark Christian Felipe R. Redillas

**Affiliations:** Department of Biology, College of Science, De La Salle University, 2401 Taft Ave., Manila 0922, Philippines; lady_tavu@dlsu.edu.ph

**Keywords:** rice (*Oryza sativa*), reactive oxygen species (ROS), oxidative stress, antioxidant defense, gene regulation, abiotic stress, stress adaptation

## Abstract

Oxidative stress, arising from environmental challenges such as drought, salinity, extreme temperatures, and pathogen attack, significantly impairs rice (*Oryza sativa*) growth, yield, and grain quality. This review provides a comprehensive synthesis of the mechanisms underlying oxidative stress in rice, with a focus on the generation of reactive oxygen species (ROS), their physiological and molecular impacts, and the antioxidant defense systems employed for mitigation. The roles of enzymatic and non-enzymatic antioxidants, along with key transcription factors, signaling pathways, and stress-responsive genes, are explored in detail. This study further highlights varietal differences in oxidative stress tolerance, emphasizing traditional, modern, and genetically engineered rice cultivars. Recent advances in breeding strategies, gene editing technologies, and multi-omics integration are discussed as promising approaches for enhancing stress resilience. The regulatory influence of epigenetic modifications and small RNAs in modulating oxidative stress responses is also examined. Finally, this paper identifies critical research gaps—including the need for multi-stress tolerance, long-term field validation, and deeper insights into non-coding RNA functions—and offers recommendations to inform the development of climate-resilient rice varieties through integrative, sustainable strategies.

## 1. Introduction

Oxidative stress is a significant physiological challenge faced by plants, particularly under environmental stress conditions. It arises when the production of ROS, such as superoxide radicals, hydrogen peroxide, and hydroxyl radicals, exceeds the plant’s antioxidant defense capacity [1,2,3]. This imbalance leads to cellular damage, disrupting metabolic processes and impairing plant growth and development [4]. Under normal conditions, plants maintain a balance between ROS generation and scavenging through enzymatic (superoxide dismutase, catalase) and non-enzymatic mechanisms (ascorbate, glutathione) [2]. However, during stress conditions like drought, salinity, extreme temperatures, and pathogen attacks, excessive production of ROS overwhelms the plant’s antioxidant systems, resulting in oxidative damage to key cellular components, including lipids, proteins, and nucleic acids [4]. Such oxidative damage not only impairs essential cellular functions but also significantly reduces crop yield, particularly in staple crops like rice (*Oryza sativa*), which are crucial for global food security.

Rice is a global crop providing food for more than half of the world’s population. Its production is increasingly threatened by various abiotic and biotic stressors, including drought, salinity, extreme temperatures, and pathogen infections [5,6,7]. These environmental stresses trigger oxidative stress in rice, negatively impacting growth, grain yield, and quality [8,9]. Understanding how rice plants respond to oxidative stress is crucial for developing strategies to enhance stress tolerance and ensure sustainable rice production in a changing climate. Recent advances in molecular biology and plant physiology have revealed complex regulatory networks governing oxidative stress responses in rice, offering new ways to improve resilience through breeding and biotechnology [10].

Rice is particularly vulnerable to oxidative stress due to its cultivation in environments prone to water scarcity, high salinity, and temperature fluctuations. Drought notably reduces yields in water-intensive crops like rice, while coastal salinity induces ionic toxicity and osmotic stress, increasing ROS production [11]. Additionally, low-temperature stress (LTS) in high-altitude regions and heavy metal contamination in soil contribute to oxidative stress, leading to reduced photosynthesis, biomass production, and grain quality [12]. The ability of rice plants to mitigate oxidative stress through adaptive responses such as upregulation of antioxidant defense systems and activation of stress-responsive genes is critical for maintaining productivity under these adverse conditions [13].

Although previous reviews have examined oxidative stress and antioxidant defense mechanisms in plants and rice, most have primarily focused on classical antioxidant pathways and general stress physiology. Few integrate recent advances in gene editing, multi-omics technologies, epigenetic regulation, and the role of small RNAs in oxidative stress responses. Moreover, varietal differences in oxidative stress tolerance among traditional, modern, and genetically engineered rice cultivars remain underexplored. This review synthesizes emerging molecular innovations and practical strategies, providing an updated, integrative perspective on breeding stress-resilient rice varieties.

Given the importance of rice in global food security and its susceptibility to oxidative stress, a comprehensive understanding of the mechanisms and adaptive responses involved is essential. This review synthesizes current knowledge on oxidative stress in rice, focusing on ROS sources and regulation, physiological impacts, and adaptive responses. It also emphasizes recent advances in breeding and biotechnological strategies, including genetic engineering and multi-omics studies, aimed at improving rice stress tolerance. Recommendations for future research are provided to guide the development of resilient rice varieties and sustainable production systems.

This review is limited to oxidative stress in rice and does not generalize to other crops. It primarily addresses stressors such as drought, salinity, extreme temperatures, and pathogens, excluding factors like pollution or nutrient deficiencies. Research gaps, particularly in long-term field studies and multi-stress environments, may limit broad agricultural applications. Nevertheless, by integrating recent advances and foundational studies, this review offers valuable insights to inform breeding programs and biotechnological innovations that enhance rice resilience. Ultimately, mitigating oxidative stress will contribute to stable yields, sustainable production, reduced reliance on external inputs, and improved food security, especially in regions increasingly impacted by climate change.

## 2. Oxidative Stress in Rice

This section examines how abiotic stressors such as drought, salinity, and extreme temperatures contribute to oxidative stress in rice by inducing excessive production of ROS [14,15]. It also discusses the physiological impacts of oxidative stress, antioxidant defense mechanisms, and recent advances in molecular and genetic regulation to enhance stress resilience.

### 2.1. Sources and Roles of Reactive Oxygen Species (ROS)

In rice, the production of ROS is a primary response to environmental stresses such as drought, salinity, heat, and pathogen attacks. Key cellular organelles—including chloroplasts, mitochondria, peroxisomes, and plasma membrane NADPH oxidases—serve as major sources of ROS under these conditions. For example, drought stress enhances photorespiration in chloroplasts, increasing ROS generation and causing oxidative damage to proteins, lipids, and DNA [4]. Peroxisomes also contribute to ROS buildup during drought, further intensifying oxidative stress [4]. Plasma membrane NADPH oxidases, encoded by genes like *OsRbohA*, are upregulated in response to heat and salinity, amplifying ROS production [16]. Although excessive ROS can be harmful, these molecules also serve important roles as signaling molecules in stress responses, programmed cell death, and interactions with phytohormone and calcium signaling pathways [4,17]. The mechanisms by which different environmental and biological stressors induce the production of ROS in rice plants are summarized in Table 1. It shows the specific organelles and the physiological responses associated with each stressor, including drought, salinity, heat stress, and pathogen interactions.

The balance between ROS production and scavenging mechanisms is sensitive, as excessive ROS can severely impair plant performance. For instance, under salinity stress, mitochondria become major sites of ROS overaccumulation, disrupting cellular metabolism and signaling, leading to mitochondrial dysfunction and reduced plant growth [14]. This imbalance highlights the complexity of oxidative stress in rice, as the severity and type of environmental stress can differentially affect cellular responses. Similarly, drought stress elevates photorespiration in chloroplasts, leading to increased ROS levels that, if uncontrolled, can result in oxidative damage and even cell death [4].

Oxidative stress is a common response to various environmental stressors, leading to the overproduction of ROS, which can damage cellular components such as proteins, lipids, and DNA [17,21,22]. Plants have evolved several mechanisms to mitigate these effects, including the activation of antioxidant enzymes and non-enzymatic antioxidants [21,22]. The photosynthetic apparatus is particularly sensitive to oxidative stress, with photosystems I and II among the most affected components [17,21,23,24]. Under stress conditions, rice plants often downregulate photosynthetic activity to limit further ROS production, a protective response observed under various environmental challenges [21,23,24].

Seedlings exposed to water deficit conditions often downregulate their photosynthetic apparatus to prevent excessive ROS production [25]. However, oxidative stress responses in rice are not confined to the seedling stage but are evident across all developmental stages, including vegetative, flowering, and grain-filling phases. At the seedling stage, oxidative stress can markedly impact growth and development, with studies showing increased sensitivity to drought and oxidative damage when key regulatory genes are suppressed [26]. During the vegetative stage, the regulation of ROS levels and antioxidant enzyme activities plays a crucial role in maintaining redox homeostasis and safeguarding photosynthetic efficiency [27]. Similarly, during the flowering and grain-filling stages, oxidative stress responses, including the activation of antioxidant pathways and stress-responsive genes such as *OsPP18*, are essential for maintaining reproductive success and grain quality under adverse conditions [26,28]. These stage-specific responses highlight the dynamic and regulated nature of oxidative stress mitigation mechanisms throughout the rice life cycle.

Similarly, salinity stress triggers significant ROS accumulation in mitochondria, further impairing energy production and inducing signaling cascades linked to oxidative damage [14,15]. Plasma membrane NADPH oxidases, particularly *OsRbohA* and *OsRbohB*, also play an important role by producing ROS during both abiotic stresses like heat and pathogen defense, showing the dual role of ROS in stress response and defense mechanisms [16,20]. This mechanism reveals that ROS is not purely negative. For instance, in response to pathogen attacks, rice cells produce ROS through NADPH oxidases, which help to combat invading pathogens like *Magnaporthe oryzae*; yet, pathogens have evolved mechanisms to scavenge host-derived ROS, complicating the defense process [20]. Thus, ROS management is a balancing act; too much ROS leads to oxidative damage, but appropriate levels contribute to stress acclimation and improved resilience.

### 2.2. ROS Scavenging and Antioxidant Defense Systems

Rice plants rely on a sophisticated antioxidant defense system composed of enzymatic and non-enzymatic components to counteract the damaging effects of ROS generated under stress conditions. Among the enzymatic antioxidants, superoxide dismutase (SOD) converts superoxide radicals into hydrogen peroxide, which is subsequently decomposed into water and oxygen by catalase (CAT) and ascorbate peroxidase (APX) [29,30]. Glutathione peroxidase (GPX) and glutathione reductase (GR) further contribute by maintaining redox homeostasis through the reduction of hydrogen peroxide and organic hydroperoxides [31]. These enzymes, whose expressions are tightly regulated by stress signals, are essential for preventing oxidative damage to cellular structures. Table 2 summarizes both enzymatic and non-enzymatic antioxidant mechanisms in rice.

A growing body of research demonstrates that the activities of key antioxidant enzymes such as SOD, CAT, and APX vary considerably under different abiotic stress conditions, including drought, salinity, and heat stress. Under drought stress, for instance, SOD activity generally increases, with drought-tolerant cultivars exhibiting significantly higher activity levels that help mitigate oxidative damage, while sensitive cultivars show less pronounced increases or even declines [33,34]. Similarly, CAT activity tends to increase in drought-tolerant cultivars, maintaining hydrogen peroxide scavenging capacity, whereas sensitive cultivars often show lower or less sustained CAT activity [33]. APX activity also rapidly increases in response to drought, particularly in tolerant cultivars [33,34]. Under salinity stress, SOD and CAT activities increase in both tolerant and sensitive cultivars, but to a greater extent in tolerant ones, aiding ROS detoxification [35]. APX activity, however, shows divergent patterns—increasing more in sensitive cultivars while remaining relatively unchanged in tolerant ones [35]. Under heat stress, all three enzymes increase, with tolerant cultivars displaying higher activity levels that contribute to greater resilience [36,37]. Overall, tolerant rice cultivars exhibit higher baseline and stress-induced activities of antioxidant enzymes, enabling more effective management of oxidative stress and cellular homeostasis under adverse conditions [33,35,37]. For example, drought-tolerant cultivars like HY73 show sustained antioxidant enzyme activity under drought, while sensitive cultivars like HHZ exhibit weaker responses and greater oxidative damage [38]. Similarly, salt-tolerant cultivars such as BRS Bojuru show significant increases in SOD and CAT activities, whereas sensitive cultivars like BRS Pampa are less effective at activating these defenses [35].

Enzymatic antioxidants are known to neutralize ROS directly; however, non-enzymatic antioxidants such as glutathione (GSH), ascorbate (vitamin C), flavonoids, and phenolic compounds act as redox buffers. These molecules scavenge free radicals and regenerate other antioxidants under stress conditions [31,32], offering additional protection, particularly during prolonged exposure to environmental stressors. Non-enzymatic antioxidants such as ascorbate (AsA), glutathione (GSH), and flavonoids play crucial roles in helping rice plants cope with various stress conditions. Ascorbate is vital for protecting the photosynthetic apparatus from oxidative damage by scavenging ROS generated during stress, thereby preserving photosystem efficiency and preventing chlorophyll degradation [39,40]. Additionally, ascorbate functions in the ascorbate–glutathione (AsA-GSH) cycle, maintaining redox homeostasis and regulating stress-responsive gene expression [41,42]. Glutathione is pivotal in detoxifying ROS, particularly hydrogen peroxide, through the AsA-GSH cycle, protecting cellular components from oxidative injury [42]. Enhanced expression of genes involved in GSH metabolism, such as glutathione synthetase (OsGS), improves redox balance and stress tolerance in rice [43]. Flavonoids contribute both to pathogen defense and oxidative stress mitigation; they act as antioxidants and antimicrobial agents, neutralizing ROS and inhibiting pathogen growth, as well as scavenging ROS under drought and heavy metal stress [40,44].

The coordinated activity of enzymatic and non-enzymatic systems is critical for maintaining cellular homeostasis and ensuring stress tolerance in rice. Together, these defense mechanisms mitigate oxidative damage and enable plants to cope with drought, salinity, and pathogen attack. However, their efficiency can be compromised under extreme stress. Despite the upregulation of antioxidant enzymes, excessive ROS accumulation may still overwhelm the system, leading to cellular damage [45]. Notably, antioxidant defense mechanisms in rice also vary across developmental stages and tissues, further influencing stress resilience. At the seedling stage, rice plants demonstrate a vigorous antioxidant response, with heightened SOD, CAT, and APX activities in both leaves and roots, particularly under stresses such as silver nanoparticles, drought, and salinity [46,47]. Roots often display higher antioxidant enzyme activities than shoots at this stage, making them the primary sites of stress mitigation. Melatonin treatments further enhance antioxidant capacity and growth during early development [48]. During the flowering stage, antioxidant defenses remain essential, especially in tissues like flag leaves and spikelets, where heat-tolerant genotypes maintain elevated antioxidant enzyme activities under heat stress, aiding reproductive success [49]. Likewise, low-temperature stress increases CAT and peroxidase (POD) activities in resistant varieties during reproduction, whereas sensitive varieties show diminished responses [12]. Tissue-specific patterns are also evident; leaves exhibit robust antioxidant responses under salinity stress, although premature leaf senescence is associated with declining SOD, CAT, and APX activities, leading to oxidative damage [50,51]. Roots consistently show higher antioxidant enzyme activities compared to leaves, underscoring their central role in stress adaptation [46].

Although genetic engineering approaches have shown that overexpression of antioxidant genes (e.g., SOD, APX) enhances stress tolerance [30], concerns remain about the long-term ecological and genetic stability of transgenic rice. Moreover, non-enzymatic antioxidants, though vital, may be limited by their regeneration capacity, highlighting the need for strategies to enhance their biosynthesis [31,32]. A significant dimension of antioxidant defense lies in its regulation. The expression and activity of antioxidants are controlled by complex signaling pathways, including transcriptional activation, redox-based modifications, and retrograde signaling [52]. Disruptions in these networks can impair the plant’s oxidative stress response. Thus, further research is needed to better understand how rice prioritizes antioxidant functions under varying environmental conditions [45].

### 2.3. Molecular and Genetic Regulation

The molecular and genetic regulation of oxidative stress responses in rice involves a complex network of signaling pathways and transcription factors that modulate ROS levels. Key components of this regulatory system include mitogen-activated protein kinase (MAPK) pathways, as well as WRKY and NAC transcription factors, which together contribute to the plant’s ability to respond to abiotic and biotic stressors [53]. Table 3 summarizes the key molecular and genetic regulators involved in oxidative stress responses in rice, including MAPK pathways, WRKY and NAC transcription factors, zinc finger proteins, and peroxidase genes. These elements modulate ROS levels and enhance stress tolerance in rice plants under diverse environmental stressors such as drought, salinity, and pathogen infection [54,55].

For instance, the MAPKKK28-MKK1-MPK1 cascade is essential for abscisic acid (ABA) signaling, influencing seed germination, root growth, and stress tolerance in rice [53]. MAPKs such as OsMPK1 are activated by wounding and pathogen attacks, while OsMAPK12-1 enhances disease resistance against bacterial pathogens but inhibits seed germination and seedling growth, illustrating the dual roles of MAPKs in balancing defense and development [61,62]. Moreover, crosstalk between MAPK pathways and transcription factors—such as the interaction between OsMKK4-OsMPK1 and OsWRKY53, is implicated in wounding signaling [61]. WRKY transcription factors, including OsWRKY50 and OsWRKY87, enhance tolerance to salt, drought, and salinity stress by interacting with ABA and stress-activated kinases [54,55]. Similarly, OsWRKY03 and OsWRKY45 contribute to pathogen resistance and drought stress adaptation through regulation of downstream defense-related genes [63]. NAC transcription factors, such as *OsNAC006* and *OsNAC022*, are critical regulators of drought, heat, and salinity stress responses, while *ONAC122* and *ONAC131* contribute to disease resistance [64,65]. These regulators modulate ROS homeostasis and stress-responsive gene expression through both ABA-dependent and independent signaling pathways.

Studies have shown that knock-out mutants of OsMPK15 exhibit increased ROS production, leading to enhanced resistance against pathogens, indicating that MAPK pathways play a balancing role in managing ROS levels and preventing oxidative damage [56]. This dual role underscores MAPKs’ importance in both promoting stress tolerance and maintaining cellular homeostasis. However, the precise mechanisms by which these pathways achieve such balance remain poorly understood. Further research could clarify how *OsMPK15* influences other stress-responsive elements, potentially offering new targets for crop improvement.

WRKY transcription factors, particularly WRKY114, also play a significant role in oxidative stress responses. Overexpression of WRKY114 in rice leads to decreased salt-stress tolerance and reduced sensitivity to abscisic acid (ABA), indicating its involvement in stress response regulation [57]. This negative impact on stress tolerance suggests that although WRKY factors are integral to signaling networks, their overactivation might trigger maladaptive responses under certain stress conditions. Therefore, studying WRKY regulation is essential to prevent unintended consequences, particularly when using genetic engineering approaches to enhance rice stress resilience. Beyond WRKY114, OsWRKY87 improves drought and salinity tolerance by interacting with SAPK10 and activating ABF1 transcription, illustrating the interconnected nature of these transcriptional regulators [55].

NAC transcription factors, such as *ONAC066*, positively contribute to oxidative stress tolerance by upregulating stress-related genes and reducing ROS accumulation. *ONAC066* binds to the *OsDREB2A* promoter, activating its transcription and enhancing drought and oxidative stress tolerance [58]. Another key gene, *OsPP18*, regulated by SNAC1, plays an important role in ROS homeostasis through ABA-independent pathways, emphasizing the role of NAC transcription factors in modulating both ABA-dependent and independent stress responses [26]. Other NAC factors like *OsNAC006* and *OsNAC022* further strengthen drought, heat, and salt tolerance by regulating stress-responsive genes [64,65]. These findings demonstrate the flexibility of NAC transcription factors in coordinating the plant’s responses to diverse environmental stress, including drought and oxidative stress.

Other crucial genes involved in ROS regulation include OsADR3, a zinc finger protein that enhances drought tolerance by increasing ROS scavenging and ABA sensitivity [59]. The *OsPRX83* gene, a peroxidase, enhances osmotic and oxidative stress tolerance through ABA-dependent pathways [60]. Both genes illustrate the importance of balancing ROS production and scavenging to maintain optimal plant growth under stress conditions. Their ability to mediate tolerance through different signaling pathways suggests potential strategies for improving rice resilience by targeting specific regulatory nodes. Importantly, the MAPK, WRKY, and NAC pathways are not isolated but exhibit substantial integration and crosstalk. For example, phosphorylation of *OsWRKY53* by *OsMPK1* and the influence of MAPKs on *OsNAC006* highlight a complex regulatory network modulating stress responses in rice [61,66,67].

**Figure 1:**
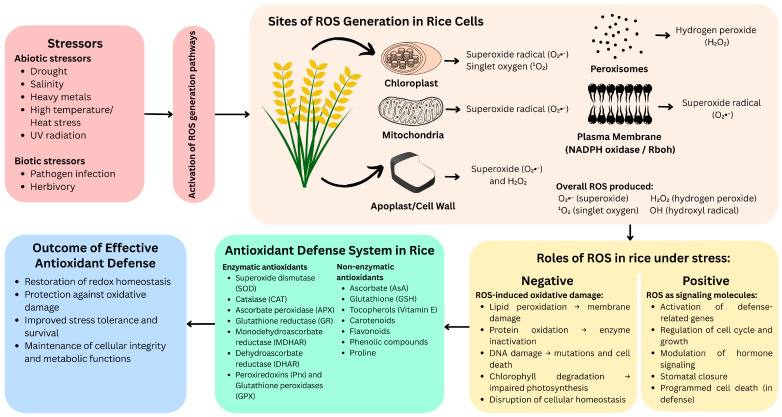
Generation, roles, and detoxification of reactive oxygen species (ROS) under oxidative stress in rice.

As summarized in Figure 1, ROS generated under various stress conditions exhibit dual functions in rice, acting as both signaling molecules and agents of cellular damage, counteracted by the antioxidant defense system. Schematic diagram illustrating the generation of reactive oxygen species (ROS), their dual roles, and the antioxidant defense system in rice (*Oryza sativa*) under oxidative stress. Abiotic and biotic stressors trigger ROS production in multiple subcellular compartments. While moderate ROS levels function in signaling to activate defense responses, excessive ROS cause oxidative damage. The rice antioxidant defense system—comprising enzymatic and non-enzymatic components—mitigates ROS accumulation, restores redox balance, and enhances stress tolerance.

## 3. Impact of Oxidative Stress on Rice Growth and Yield

### 3.1. Effects on Plant Physiology and Biochemistry

The impacts of oxidative stress affect a number of physiological processes in rice, including photosynthesis, respiration, and nutrient uptake, influencing plant growth and yield. Oxidative damage to chloroplast structures, triggered by excess iron and intense light, disrupts photosynthesis by reducing chlorophyll content and impairing energy conversion efficiency [8,68]. This damage, even before visible symptoms appear, emphasizes the sensitivity of photosynthetic machinery to oxidative stress, ultimately compromising crop productivity [69]. The role of oxidative stress in respiration is equally significant. Under drought conditions, photorespiration increases, leading to the excessive production of ROS in chloroplasts and mitochondria, which causes severe cellular damage, including lipid peroxidation and protein oxidation [4]. This not only affects respiration efficiency but also compromises the plant’s energy balance, reducing overall growth and development. Table 4 shows the impact of oxidative stress on key physiological processes in rice, including photosynthesis, respiration, nutrient uptake, stomatal conductance, and lipid peroxidation.

Nutrient uptake, a critical aspect of plant growth, is also negatively influenced by oxidative stress. For example, phosphorus and potassium deficiencies disrupt nitrogen metabolism, and water stress worsens oxidative damage, further impairing the plant’s ability to absorb and utilize nutrients effectively [70,71]. As oxidative stress progresses, it extends to other physiological processes, such as stomatal conductance and water retention, leading to decreased turgor pressure and reduced plant growth, especially under conditions of drought and high temperatures [72]. Markers such as malondialdehyde, which signal lipid peroxidation, reflect the extent of oxidative damage and show the biochemical toll on rice plants [8,73].

The effects of oxidative stress on photosynthesis, respiration, and nutrient uptake are well documented, but there is still a need to focus on mitigation strategies. The overexpression of certain genes, such as *OsDJ-1C*, has shown promise in improving root architecture, enhancing photosynthetic efficiency, and boosting abiotic stress tolerance by modulating antioxidant defense and redox homeostasis [74]. Furthermore, research into drought- and heat-tolerant rice varieties is crucial, as these can improve seed setting and yield production under oxidative stress [72]. Since oxidative stress undeniably decreases rice productivity, advancements in genetic improvement and advanced understanding of the physiological and biochemical responses to abiotic stress are imperative as they offer ways to enhance stress tolerance and secure food production under adverse conditions.

### 3.2. Impact on Grain Yield and Quality

Oxidative stress, driven by abiotic factors like drought, salinity, and high temperatures, plays a critical role in reducing rice yield and quality by disrupting key physiological processes. Grain yield, particularly in terms of size and number, is severely impacted under oxidative stress conditions due to the overproduction of ROS, which damages cellular structures and interferes with metabolic pathways essential for growth [75]. Specifically, the flowering stage is highly vulnerable, where high temperatures result in significant yield losses. Oxidative stress affects grain quality, reducing starch deposition during the grain-filling period and increasing grain chalkiness, which compromises the nutritional and market value of rice [76]. Table 5 shows the overview of how oxidative stress and various abiotic factors such as drought, high temperature, and salinity affect rice yield and grain quality. It also details potential mitigation strategies, from short-term interventions like the application of antioxidants and nanoparticles to long-term approaches such as breeding stress-tolerant rice varieties. This synthesis emphasizes the need for continued research into genetic improvements and molecular interventions to enhance rice resilience.

On a biochemical level, oxidative stress leads to lipid peroxidation and protein oxidation, degrading grain quality and rice’s nutritional properties [81]. These physiological disruptions emphasize the importance of cellular defense mechanisms, including enzymatic and non-enzymatic antioxidants, which play a key role in mitigating oxidative damage [8]. Despite these defenses, the stress imposed by unfavorable environmental conditions often overwhelms the plant’s protective systems, leading to compromised yield and quality. The effects of oxidative stress show the urgent need for both short- and long-term mitigation strategies. Immediate interventions, such as applying antioxidants like cobalt or using nanoparticles like chitosan–magnesium oxide (CMgO), have shown promise in improving yield and grain quality under stress conditions [80]. However, these solutions only offer temporary relief and are often impractical on scale. More sustainable approaches, such as genetic improvements and breeding stress-tolerant rice varieties, respond to oxidative stress. The development of drought and salt-tolerant cultivars has already demonstrated increased resilience to oxidative stress, suggesting that further advancements in this area could secure rice productivity against climate-induced stress [18,72]. Current research advances in understanding the molecular mechanisms behind oxidative stress tolerance. Omics technologies, such as RNA sequencing and genome-wide SNP analysis, have identified key genes and metabolites that regulate stress responses, paving the way for the genetic modification of rice for improved stress tolerance [18]. However, these approaches come with challenges, including ethical considerations surrounding genetically modified organisms (GMOs) and the potential environmental impacts of deploying such technologies at scale.

### 3.3. Variations Among Different Rice Varieties

The variation in oxidative stress responses among rice varieties is a key factor in determining their resilience and productivity under stress conditions. Table 6 shows the comparative analysis of oxidative stress tolerance traits across traditional, modern, and genetically engineered rice varieties.

Traditional varieties, such as Co13, have shown strong tolerance to oxidative stress, due to their efficient photosystem II function and low ion leakage, which indicates better membrane integrity under stress [82]. The Korgut variety has higher levels of unsaturated fatty acids compared to the Jaya variety, which enhances membrane fluidity, allowing for it to better withstand salinity-induced oxidative stress [83]. These inherent traits show the evolutionary adaptation of traditional varieties to their local environments, making them valuable in areas prone to specific abiotic stresses such as salinity or drought. On the other hand, modern varieties have been selectively bred for traits like oxidative stress tolerance, often making them comparable or even superior to traditional cultivars in stress resilience. High-yielding modern cultivars like Swarnaprabha and Kattamodan have demonstrated enhanced drought recovery, increased membrane stability, and sensitive antioxidant enzyme activities, all of which contribute to their better performance under oxidative stress conditions [84]. This selective breeding emphasizes the importance of oxidative stress tolerance as a key trait in modern rice production, ensuring both yield stability and stress resilience in environments affected by climate change. Although both traditional and modern varieties exhibit significant tolerance to oxidative stress, modern varieties have been refined through breeding programs to optimize these traits. For instance, modern japonica cultivars like Koshihikari and indica varieties such as IR58 show similar resilience to oxidative damage as traditional varieties but benefit from the focused selection of stress-tolerant traits during their development [82]. These improvements reflect the shift in rice breeding programs, which increasingly prioritize abiotic stress resistance to maintain productivity in changing environmental conditions.

Genetically engineered varieties further extend the capacity of rice to withstand oxidative stress by introducing specific genes that enhance antioxidant responses. Varieties like Binadhan-11 and BRRI dhan52, which carry the *SUB1* gene, exhibit superior antioxidant enzyme activities, particularly ascorbate peroxidase (APX) and peroxidase (POD), enabling them to better tolerate osmotic stress compared to non-*SUB1* varieties [85]. This genetic modification represents a significant advancement in rice cultivation, as these varieties not only maintain higher yields under stress but also exhibit enhanced resilience to environmental fluctuations. However, the use of genetically modified organisms (GMOs) in agriculture raises ethical and environmental concerns, particularly regarding their long-term ecological impacts and the socio-economic implications for farmers. The identification of key genes and metabolites responsible for oxidative stress tolerance is crucial for breeding drought- and salt-tolerant varieties, which will be essential for maintaining rice production in stress-prone areas [18]. As environmental conditions continue to shift, developing rice cultivars with enhanced stress tolerance through traditional, modern, and biotechnological methods will be necessary for ensuring food security and agricultural sustainability.

## 4. Adaptive Responses to Oxidative Stress in Rice

### 4.1. Physiological and Biochemical Adaptations

Physiological and biochemical adaptations play a vital role in rice’s ability to mitigate oxidative stress, particularly through mechanisms such as osmolyte accumulation, proline synthesis, and enhanced antioxidant enzyme activity [9]. These responses help reduce the harmful effects of ROS, maintain cellular homeostasis, and promote plant survival under adverse conditions. Table 7 provides an overview of these key mechanisms, including osmolyte accumulation, proline synthesis, antioxidant enzyme activity, and the regulation of calcium-dependent protein kinases (CPKs) [9,86].

A central physiological adaptation is the accumulation of osmolytes such as proline and glycine betaine, which facilitate osmotic adjustment and protect cellular components from ionic toxicity and oxidative damage. In addition to preserving membrane integrity, osmolytes function as ROS scavengers, contributing to redox balance under stress [9,86,87,88]. Besides proline, other osmolytes such as sugars and polyamines also accumulate during drought and salinity stress, reinforcing both osmotic adjustment and ROS mitigation [9].

Proline synthesis is particularly crucial in rice’s stress response, functioning both as an osmoprotectant and antioxidant [9,93]. Under oxidative stress, proline accumulation is regulated by the upregulation of proline biosynthesis genes such as *OsP5CS* and *OsP5CR*, alongside the downregulation of catabolic genes like *OsPRODH* [86,87]. Transcriptional control of proline metabolism, such as the activation of *OsP5CR* by *OsMADS25*, further enhances proline accumulation and stress tolerance [94]. This dual role of proline—in osmotic balance and ROS scavenging—highlights its significance in stress resilience and makes it a strategic target for developing stress-tolerant rice cultivars through genetic improvement.

Drought-tolerant rice varieties typically exhibit heightened antioxidant enzyme activity, which protects plants by neutralizing ROS before they inflict cellular damage [8,9,10,91,92]. The activities of enzymatic antioxidants, including superoxide dismutase (SOD), catalase (CAT), peroxidase (POD), and ascorbate peroxidase (APX), are significantly upregulated under stress, sustaining redox equilibrium [9,86]. Additionally, non-enzymatic antioxidants such as ascorbate and glutathione also play crucial roles in mitigating oxidative damage [95].

Calcium-dependent protein kinases (CPKs) further contribute to regulating antioxidant defenses by activating stress-responsive genes and antioxidant enzymes [30,76]. Their involvement in both ROS homeostasis and signal transduction underscores their potential as molecular targets for engineering stress-resilient rice cultivars [30].

Exogenous application of compounds like salicylic acid (SA) has also been demonstrated to enhance antioxidant enzyme activity, offering an agronomic approach to bolster rice stress tolerance under field conditions [96]. Similarly, nitric oxide (NO) signaling plays a key role in improving antioxidant capacity and reducing oxidative damage; application of NO donors such as sodium nitroprusside (SNP) enhances antioxidant defenses and suppresses ROS accumulation [97]. While these physiological and biochemical mechanisms are effective in improving stress tolerance, their efficiency varies among rice cultivars and environmental conditions. Although genetic engineering strategies that enhance osmolyte synthesis and antioxidant activity offer significant potential for improving rice resilience [87], they may also pose risks to genetic diversity and ecosystem balance. Therefore, sustainable rice cultivation must carefully balance the benefits of genetic modifications with the conservation of the inherent adaptability of traditional rice varieties.

### 4.2. Molecular and Genetic Adaptations

Molecular and genetic adaptations to oxidative stress in rice involve a complex interaction of key genes, quantitative trait loci (QTLs), and molecular pathways that contribute to stress tolerance. Table 8 summarizes the molecular and genetic adaptations of rice to oxidative stress, focusing on key genes, quantitative trait loci (QTLs), and regulatory pathways.

A primary focus in this field has been identifying genes such as *sub1A*, which is primarily linked to submergence tolerance. It also plays an indirect role in managing oxidative stress by enhancing antioxidation pathways [98]. This connection between submergence and oxidative stress suggests that rice plants employ overlapping stress responses, where adaptations to one stressor may confer resistance to others. The Saltol QTL, containing the transcription factor *OsHBP1b*, further exemplifies how molecular components can enhance salinity and drought tolerance by modulating antioxidant enzyme activities and reducing ROS levels [99]. These molecular responses not only point to natural mechanisms of resilience but also offer potential genetic targets for crop improvement programs focused on increasing rice’s ability to withstand oxidative stress. Transcription factors such as OsGRAS23 also play a significant role in enhancing drought and oxidative stress tolerance by regulating the expression of stress-responsive genes and minimizing H_2_O_2_ accumulation [100]. The involvement of these transcription factors shows the importance of gene regulation in stress adaptation, providing pathways that could be targeted for biotechnological interventions. Genetic modifications, such as the overexpression of OsPRX83 (a peroxidase precursor gene), have been shown to enhance osmotic and oxidative stress tolerance in rice, offering a practical approach for developing rice varieties that can thrive in challenging environmental conditions [60].

At the molecular level, rice plants employ an advanced network of regulatory components, including protein kinases, phosphatases, and transcription factors, to maintain ROS homeostasis and activate stress resistance mechanisms [30]. This regulatory network is crucial for fine-tuning the plant’s response to oxidative stress, allowing for it to balance growth and survival under adverse conditions. Furthermore, pathways involving polyamine and proline synthesis also play a crucial role in modulating antioxidant activity, with polyamines acting as ROS scavengers and stabilizers of membrane integrity under oxidative stress [98]. This reveals a complex relationship between biochemical pathways and molecular regulation, where metabolic adjustments reinforce the plant’s defense systems. In terms of QTLs, the sub1A locus is a notable contributor to oxidative stress tolerance, particularly in genotypes bred for submergence resistance [98]. The identification of such QTLs offers valuable insights for rice breeding programs aiming to develop multi-stress-tolerant varieties. Molecular studies have identified over 50 differentially expressed transcription factors in contrasting rice genotypes under oxidative stress, emphasizing the genetic diversity present within rice species and the potential for utilizing this diversity in breeding for stress tolerance [101]. Yet, this approach also poses a challenge: the potential trade-offs between increasing stress tolerance and maintaining desirable traits such as yield and grain quality. For instance, although certain genetic modifications can enhance oxidative stress tolerance, they may accidentally affect other growth-related pathways, raising concerns about long-term agricultural sustainability.

### 4.3. Role of Epigenetics and Small RNAs

The role of epigenetics and small RNAs, particularly microRNAs (miRNAs), in regulating oxidative stress responses in rice emphasizes the elaborate mechanisms of gene expression modulation that allow for plants to adapt to environmental stressors. Table 9 shows the role of epigenetic modifications and miRNAs in regulating oxidative stress responses in rice, the pathways involved, their functions in stress adaptation, and examples from recent research.

Epigenetic modifications, including RNA-directed DNA methylation (RdDM), histone modifications, and the activity of small RNAs, are crucial in coordinating the plant’s response to oxidative stress. The RdDM pathway helps regulate gene expression by silencing transposable elements, maintaining genome stability under stress conditions, and ensuring proper developmental processes [81,102]. This dynamic system is not only critical for stress adaptation but also offers a mechanism for the plant to adjust its genetic responses to fluctuating environmental conditions. However, the complexity of these epigenetic processes raises important questions about the long-term impacts of these modifications, particularly in how they influence plant development and stress memory across generations. One aspect of epigenetic regulation involves histone modifications, which facilitate chromatin remodeling, thereby controlling access to stress-responsive genes. Under salt stress, for example, rice plants exhibit genome-wide changes in H3K27 modifications, which promote nucleosomal rearrangement and allow for transcription factors to access previously inaccessible loci [103]. This regulatory flexibility is pivotal in enabling rapid responses to oxidative stress.

In addition to epigenetic modifications, miRNAs have emerged as central regulators in oxidative stress adaptation. miRNAs such as *miR156*, *miR166*, and *miR171* are differentially expressed under oxidative stress, modulating the expression of genes that manage ROS scavenging and homeostasis [104]. Specifically, *miR156* targets genes like *OsSPL2* and *OsTIFY11b*, which are involved in regulating defense-related responses under oxidative stress, showcasing miRNAs as pivotal players in rice’s molecular defense strategies [104]. Another example is *miR172*, which contributes to ROS scavenging under salt stress, further demonstrating miRNAs’ role in maintaining oxidative balance [105]. These findings show miRNAs as potential genetic tools for enhancing oxidative stress tolerance, offering breeders a way to improve rice varieties’ resilience. However, the manipulation of miRNA pathways and histone modification brings up concerns regarding unintended consequences, such as off-target effects that could alter other vital processes, such as growth regulation and reproductive development.

Beyond their immediate roles in stress responses, miRNAs exhibit developmental stage-specific expression, indicating that their regulatory functions are tuned to the plant’s growth phase. For instance, *Osa-miR156j* displays differential expression across developmental stages, suggesting a role not only in oxidative stress response but also in broader developmental regulation [42]. This dual functionality reflects the adaptability of miRNAs in responding to both internal and external signals, making them a key point for future research in rice stress biology. However, the roles of miRNAs across developmental stages present challenges in their application. Modifying miRNA expression to enhance stress tolerance could potentially disrupt their regulatory functions in other critical processes, such as flowering or seed development, stressing the need for a balanced approach in using these molecular tools for crop improvement.

The interaction between epigenetic modifications and small RNAs shows a multi-layered regulatory network that governs rice’s response to oxidative stress. Stress-induced epimutations, such as DNA methylation changes, may even be inherited across generations, suggesting that rice plants could pass on stress tolerance traits epigenetically [107]. This transgenerational inheritance of stress tolerance, however promising, also prompts ethical and ecological considerations. The potential for long-term changes in the genome due to stress-induced epimutations raises questions about the stability and predictability of these modifications in future generations, especially in natural ecosystems. Moreover, while epigenetic modifications and miRNAs offer powerful tools for improving rice stress tolerance, their manipulation must be approached with caution to avoid unintended disruptions to the plant’s natural growth and development cycles.

### 4.4. Recent Advances in Breeding and Biotechnology for Oxidative Stress Tolerance

Recent advances in breeding and biotechnology have significantly enhanced rice’s oxidative stress tolerance by integrating conventional and molecular approaches to address various environmental challenges. Table 10 shows recent advances in conventional breeding and molecular biotechnology approaches, such as gene overexpression, MAS, CRISPR/Cas9, and multi-omics integration, that are being applied to enhance oxidative stress tolerance in rice.

On the biotechnological front, the identification and overexpression of key stress-responsive genes have sustained rice’s ability to mitigate oxidative damage. For instance, transgenic rice overexpressing OsDhn1 has shown greater resilience to drought, salinity, and oxidative stress by improving the plant’s capacity to scavenge ROS [108]. Such gene-based strategies complement traditional marker-assisted selection (MAS) by introducing specific genetic modifications directly linked to stress adaptation. Additionally, treatments with abscisic acid (ABA) have been found to enhance stress tolerance by activating antioxidant enzyme activities and maintaining cellular homeostasis under stress conditions [11]. However, biotechnological approaches like these offer promising solutions yet still face challenges related to regulatory, ethical, and public acceptance, particularly in regions resistant to genetically modified organisms (GMOs). The potential ecological impacts of transgenic crops also require careful consideration.

Recent gene-editing technologies, particularly CRISPR/Cas9, have emerged as critical tools for enhancing oxidative stress tolerance in rice. CRISPR/Cas9’s precision allows for targeted modifications in genes responsible for stress responses, although concerns about off-target effects and environmental impacts persist [108]. For instance, genes such as *SUB1A* for submergence tolerance and *SalTol* for salinity tolerance have been integrated into MAS to develop more stress-resilient rice varieties [85,99]. Meanwhile, transcriptomics has provided insights into the expression profiles of genes involved in oxidative stress responses, such as respiratory burst oxidase homologs (Rbohs) and brassinosteroid biosynthesis genes, which play vital roles in salinity stress tolerance [45]. This information supports the identification of stress-related genes, aiding genomic breeding efforts. Proteomics and metabolomics further deepen our understanding by uncovering the functional proteins and metabolic pathways involved in stress responses. Studies have identified key enzymes like NADP-malic enzyme and antioxidants such as glutathione and ascorbate, which are critical for managing oxidative stress [11]. Metabolomics has also uncovered protective osmolytes and antioxidants in stress-tolerant rice, providing biochemical markers for breeding [109]. The integration of multi-omics data, including genomics, transcriptomics, proteomics, and metabolomics, offers a comprehensive understanding of oxidative stress tolerance. This whole approach has led to the identification of master regulators, such as specific transcription factors, which could be targeted for genetic manipulation [101]. Multi-omics approaches have provided powerful tools for understanding the molecular mechanisms behind stress tolerance, but translating these insights into field applications remains a challenge. Traits must be validated under diverse environmental conditions to ensure their effectiveness. Ethical and socio-economic concerns, particularly related to CRISPR/Cas9 and its impact on biodiversity and food security, must also be addressed. Nonetheless, integrating multi-omics data offers promising pathways to enhance rice resilience to climate variability, supporting the sustainable cultivation of rice under adverse environmental conditions [110].

## 5. Challenges and Future Directions

Despite the advancements in understanding oxidative stress in rice, significant knowledge gaps remain, particularly in areas that are essential for enhancing the stress tolerance of rice under real-world conditions. These gaps emphasize the need for further exploration in several key domains, including the role of non-coding RNAs, long-term field validation of laboratory findings, and the development of multi-stress tolerance. While substantial progress has been made in identifying the genetic and molecular mechanisms underlying rice’s response to oxidative stress, the role of non-coding RNAs (ncRNAs) in regulating these processes remains poorly understood. ncRNAs, such as microRNAs (miRNAs) and long non-coding RNAs (lncRNAs), are known to play crucial roles in gene expression regulation, particularly in response to environmental stressors [111].

Most research on oxidative stress in rice has been conducted under controlled laboratory conditions, where environmental variables can be tightly managed. Although these studies provide valuable insights, the real challenge lies in translating these findings to the field, where rice plants are exposed to a combination of unpredictable stress over extended periods. Long-term field trials are essential to validate the effectiveness of oxidative stress mitigation strategies, such as antioxidant priming and genetic modifications, under diverse climatic and soil conditions. Research should focus on the durability of stress tolerance traits across multiple growing seasons and geographies to ensure that these strategies can be reliably implemented in large-scale agriculture [112]. Without field validation, the potential benefits of laboratory breakthroughs may remain limited in practical applications.

In the natural environment, rice plants are rarely subjected to oxidative stress in isolation. Abiotic stresses such as drought, salinity, and extreme temperatures often occur simultaneously, compounding the oxidative damage and complicating the plant’s response mechanisms. Research has mostly focused on individual stress factors, but there is a critical need to study the combined effects of multiple stresses and how rice plants can be engineered or bred for multi-stress tolerance. This includes understanding how oxidative stress pathways interact with other stress response systems, such as drought-induced stomatal closure or salinity-induced ion toxicity. Future research should aim to develop rice varieties that are not only tolerant to one stressor but can also withstand a combination of stresses, showing the complex challenges faced in real-world agricultural settings.

## 6. Conclusions and Recommendations

This systematic review provides a comprehensive overview of the implications of oxidative stress in rice and its critical relevance to sustainable agriculture. The key findings demonstrate that oxidative stress, primarily triggered by abiotic factors such as drought, salinity, and extreme temperatures, leads to the excessive accumulation of ROS, causing substantial cellular damage that impairs growth and reduces yield. Rice plants possess a sophisticated antioxidant defense system comprising both enzymatic and non-enzymatic components, which is essential for mitigating oxidative damage. The review highlights notable variability in oxidative stress tolerance among rice cultivars, with modern varieties generally exhibiting greater resilience compared to traditional landraces. This variability underscores the importance of incorporating oxidative stress management strategies into breeding programs aimed at improving rice productivity and sustainability. Furthermore, this review identifies critical knowledge gaps, particularly in understanding the role of non-coding RNAs, the necessity for long-term field validation of laboratory findings, and the complex interactions between multiple abiotic stressors that affect rice production. Addressing these gaps is essential to translate current research into practical, scalable solutions that can effectively enhance rice resilience in the context of evolving environmental challenges.

Based on the key findings and identified research gaps, the following recommendations are proposed to advance research and improve oxidative stress management in rice cultivation.

- Prioritize research on non-coding RNAs to elucidate their regulatory roles in oxidative stress response pathways, facilitating the development of novel post-transcriptional strategies to enhance stress tolerance.
- Conduct long-term field trials under diverse agro-ecological conditions to validate laboratory findings and ensure the durability and real-world applicability of stress tolerance traits across multiple growing seasons.
- Develop rice varieties with multi-stress tolerance by investigating the interactions between oxidative stress and other abiotic stressors, such as drought and salinity, to produce cultivars capable of thriving under simultaneous environmental stresses.
- Employ a systems biology approach that integrates genomics, proteomics, and metabolomics to deepen understanding of complex stress response networks and identify novel targets for breeding programs.
- Integrate oxidative stress resilience with other key agronomic traits, such as nutrient use efficiency and climate adaptability, within breeding programs aligned with climate-smart agriculture principles to enhance the sustainability of rice production systems.
- Foster interdisciplinary collaboration among researchers, plant breeders, agronomists, and policymakers to scale up the adoption of stress-tolerant rice varieties and implement effective oxidative stress mitigation strategies in support of global food security.

Implementing these strategies will not only strengthen rice resilience to oxidative stress but also contribute to sustainable agricultural practices and global food security in the face of growing environmental challenges.

## Figures and Tables

**Table 1:** Oxidative stress mechanisms in rice under various environmental stress conditions.

Stress Condition	Mechanism of ROS Production	Impact	References
Drought	Increased ROS production in chloroplasts, mitochondria, and peroxisomes due to enhanced photorespiration and impaired antioxidant defenses.	This leads to oxidative damage due to elevated ROS levels, disrupting photosynthesis and metabolic balance.	[4,18]
Salinity	Induces ROS production in mitochondria, contributing to oxidative stress and programmed cell death (PCD).	ROS accumulation damages cellular components, leading to growth inhibition and cell death.	[14]
Heat	Heat stress triggers ROS production, regulated by proteins like *OsEDS1*, which promote hydrogen peroxide scavenging to balance ROS homeostasis.	Excess ROS causes cellular damage, reduced yield, and heat-induced oxidative stress.	[19]
Pathogens	ROS generated by NADPH oxidases like *OsRbohB* during rice–*Magnaporthe oryzae* interactions as a defense response. The pathogen scavenges host-derived ROS to facilitate infection.	Host ROS combat pathogens, but excessive ROS can also lead to tissue damage and aid pathogen infection.	[20]

**Table 2:** Antioxidant defense mechanisms in rice plants under oxidative stress.

Antioxidant Defense Mechanisms	Function	References
Enzymatic Antioxidants		
Superoxide Dismutase (SOD)	Converts superoxide radicals to hydrogen peroxide	[29,30]
Catalase (CAT)	Decomposes hydrogen peroxide to water and oxygen	[29]
Ascorbate Peroxidase (APX)	Reduces hydrogen peroxide using ascorbate	[29,30]
Glutathione Peroxidase (GPX)	Reduces hydrogen peroxide and organic hydroperoxides	[29,31]
Glutathione Reductase (GR)	Regenerates reduced glutathione	[29,31]
Non-Enzymatic Antioxidants		
Glutathione (GSH)	Directly scavenges ROS and regenerates other antioxidants	[29,31]
Ascorbate (Vitamin C)	Scavenges ROS and acts as a substrate for APX	[29,31]
Phenolic Compounds, Flavonoids, Tocopherols	Contribute to overall antioxidant capacity	[32]

**Table 3:** Molecular and genetic regulation of oxidative stress in rice.

Regulatory Element	Key Findings	Impact on Stress Tolerance	References
MAPK Pathway (*OsMPK15*)	*OsMPK15* knock-out mutants exhibit increased ROS production, leading to enhanced pathogen resistance	Balances ROS levels, promotes disease resistance, and maintains cellular homeostasis.	[56]
WRKY Transcription Factors (*WRKY114*)	Overexpression of *WRKY114* decreases salt-stress tolerance and reduces sensitivity to ABA	Regulates stress responses, but overactivation can lead to maladaptive responses.	[57]
NAC Transcription Factors (*ONAC066*)	*ONAC066* activates *OsDREB2A*, enhancing drought and oxidative stress tolerance	Positively regulates stress-related genes, reduces ROS accumulation, and enhances tolerance.	[58]
Zinc Finger Protein (*OsADR3*)	*OsADR3* enhances drought tolerance by increasing ROS scavenging and ABA sensitivity	Improves ROS scavenging and stress tolerance, particularly under drought conditions.	[59]
Peroxidase Gene (*OsPRX83*)	*OsPRX83* enhances osmotic and oxidative stress tolerance via ABA-dependent pathways	Contributes to tolerance of osmotic stress and oxidative damage.	[60]
General Stress-Related Genes	Transgenic approaches (SOD and APX overexpression) have improved stress tolerance in lab settings	Enhance antioxidant activity and stress resilience.	[30]

**Table 4:** Impact of oxidative stress on physiological processes in rice.

Physiological Process	Effects of Oxidative Stress	Impact	References
Photosynthesis	Oxidative damage to chloroplast structures, reducing chlorophyll content and energy conversion efficiency, especially under excess iron and intense light.	Compromised photosynthesis leads to reduced crop productivity.	[8,68,69]
Respiration	Increased ROS production during photorespiration under drought conditions, causing lipid peroxidation and protein oxidation in chloroplasts and mitochondria.	Cellular damage affects respiration efficiency and disrupts the plant’s energy balance, limiting growth, and development.	[4]
Nutrient Uptake	Phosphorus and potassium deficiencies impair nitrogen metabolism; oxidative damage increases by water stress, limiting the plant’s ability to absorb and utilize nutrients effectively.	Impaired nutrient uptake reduces plant growth, particularly under drought and heat stress conditions.	[70,71]
Stomatal Conductance	Decreases stomatal conductance and water retention, reducing turgor pressure under drought and high temperatures.	Lower stomatal conductance leads to decreased water uptake and reduced growth, particularly during prolonged drought stress.	[72]
Lipid Peroxidation (Marker)	Malondialdehyde accumulation signals oxidative damage in cellular membranes, reflecting lipid peroxidation and the biochemical toll on the plant tissues.	Lipid peroxidation indicates the extent of oxidative damage, affecting cell integrity and overall plant health.	[8,73]

**Table 5:** Impact of oxidative stress on rice yield and quality.

Stress Factor	Impact on Yield	Impact on Quality	Mitigation
Drought	Reduced grain size and number [75] compromises the energy balance and growth of rice plants [4].	Negatively impacts the nutrient uptake [70,71].	The application of cobalt has shown promise [77], and the development of stress-tolerant rice varieties has been identified as a sustainable approach [72,78].
High Temperature	During critical stages, particularly flowering, results in significant yield losses [79].	Poor starch deposition and increased chalkiness during the grain-filling period lead to decreased nutritional value [76].	Genetic improvements aimed at enhancing stress tolerance [18] and applying antioxidants to improve the effects of oxidative stress [80].
Salinity	Contributes to a reduction in overall yield, due to the adverse effects on physiological processes [81].	The quality of rice is compromised by oxidative damage to proteins and lipids, which undermines its nutritional properties [81].	The use of CMgO nanoparticles has emerged as a promising mitigation strategy for salinity stress [80].

**Table 6:** Variation across traditional, modern, and genetically engineered varieties.

Rice Variety	Oxidative Stress Tolerance Traits	Examples
Traditional Varieties	Exhibit natural resilience to oxidative stress due to evolutionary adaptations like better membrane integrity and low ion leakage, enabling them to maintain photosystem II function under stress [82]. Higher levels of unsaturated fatty acids improve membrane fluidity, especially under salinity-induced stress [83].	Co13 [82], Korgut [83]
Modern Varieties	Selected for enhanced oxidative stress tolerance with improvements in antioxidant enzyme activities, membrane stability, and drought recovery [84].	Swarnaprabha, Kattamodan [84]
Genetically Engineered Varieties	Modified to introduce specific stress-tolerant genes, such as *SUB1*, which improve antioxidant defenses (ascorbate peroxidase and peroxidase activity), thus increasing resilience to oxidative stress and maintaining higher yields under adverse conditions [85].	Binadhan-11, BRRI dhan52 [85]

**Table 7:** Physiological and biochemical adaptations to oxidative stress in rice.

Adaptive Mechanism	Function in Stress Response	References
Osmolyte Accumulation	Accumulation of osmolytes such as proline and glycine betaine aids in osmotic adjustment, protects cellular components from ionic toxicity, and scavenges ROS. These compounds help maintain membrane integrity and balance the cellular redox environment under oxidative stress.	[87,88]
Proline Synthesis	Proline acts as both an osmoprotectant and an antioxidant. Proline synthesis genes (*OsP5CS* and *OsP5CR*) are upregulated, while proline catabolic genes (*OsPRODH*) are downregulated during oxidative stress. This regulation helps rice maintain water absorption and minimize physiological damage.	[89,90]
Antioxidant Enzyme Activity	Antioxidant enzymes such as superoxide dismutase (SOD), catalase (CAT), and peroxidase (POD) neutralize ROS, reducing oxidative damage to cellular structures. Increased enzyme activity correlates with higher stress tolerance.	[91,92]
Calcium-Dependent Protein Kinases (CPKs)	CPKs regulate antioxidant enzyme activity, improving the plant’s ability to cope with oxidative stress by modulating cellular ROS scavenging.	[76]

**Table 8:** Molecular and genetic adaptations to oxidative stress in rice.

Adaptive Mechanism	Function in Oxidative Stress Response	Examples	References
Submergence and Oxidative Stress Tolerance	Primarily involved in submergence tolerance, sub1A indirectly enhances antioxidation pathways, suggesting overlapping stress responses to submergence and oxidative stress.	Sub1A provides dual tolerance to both submergence and oxidative stress in rice cultivars.	[98]
Salinity and Drought Tolerance	Saltol QTL and OsHBP1b transcription factor enhance salinity and drought tolerance by modulating antioxidant enzyme activities and reducing ROS levels.	Saltol QTL enhances rice’s resilience to drought and salinity, offering better stress tolerance.	[99]
Transcriptional Regulation	Enhances drought and oxidative stress tolerance by regulating stress-responsive genes and reducing H_2_O_2_ accumulation, minimizing oxidative damage.	OsGRAS23 transcription factor improves rice’s adaptive responses under drought and oxidative stress.	[100]
Antioxidant Enzyme Activity	Overexpression of *OsPRX83* increases osmotic and oxidative stress tolerance by boosting ROS scavenging and antioxidant defenses.	*OsPRX83* overexpression leads to increased oxidative stress resistance in genetically modified rice.	[60]
Regulatory Components	These regulatory components maintain ROS homeostasis and activate stress resistance mechanisms, fine-tuning the plant’s response to oxidative stress.	Regulatory networks involving kinases and transcription factors modulate ROS balance under stress.	[30]
Polyamine and Proline Synthesis	Polyamines act as ROS scavengers and stabilize membrane integrity, providing protection against oxidative damage.	Polyamines contribute to antioxidant activity and stress tolerance.	[98]
Differential Gene Expression	Transcription factors modulate the plant’s response to oxidative stress, with variations across different rice genotypes revealing genetic diversity in stress tolerance.	Diverse genetic responses in rice to oxidative stress show potential for breeding stress-tolerant varieties.	[101]

**Table 9:** Epigenetic modifications and miRNA-mediated regulation of oxidative stress in rice.

Adaptive Mechanism	Function in Oxidative Stress Response	Examples
RNA-directed DNA Methylation (RdDM)	Regulates gene expression by silencing transposable elements and maintaining genome stability under stress, crucial for development and stress adaptation [81,102].	Silencing of transposable elements to maintain genome stability during stress [81].
Histone Modifications	Facilitates chromatin remodeling, controlling access to stress-responsive genes, allowing for rapid responses to oxidative stress [103].	Genome-wide changes in H3K27 modifications enable transcription factor access under stress [103].
MicroRNAs (miRNAs)	miRNAs regulate gene expression under oxidative stress by modulating ROS scavenging and homeostasis [104,105].	miR156 targets OsSPL2 and OsTIFY11b to manage ROS and oxidative stress [104]; miR172 scavenges ROS under salt stress [105].
Developmental Stage-Specific miRNA Expression	miRNAs exhibit stage-specific expression, modulating both stress response and development, reflecting their dual role in rice’s growth phases [106].	Osa-miR156j is differentially expressed across developmental stages, influencing both stress responses and development [106].
Transgenerational Epigenetic Inheritance	Stress-induced DNA methylation changes may be inherited across generations, allowing for rice plants to pass on stress tolerance traits epigenetically [107].	Transgenerational inheritance of stress tolerance traits via DNA methylation [107].

**Table 10:** Recent advances in breeding and biotechnology for enhancing oxidative stress tolerance in rice.

Approach	Function in Improving Oxidative Stress Tolerance	Examples/Findings
Gene Overexpression	Enhances drought, salinity, and oxidative stress tolerance by improving ROS scavenging ability.	Transgenic rice overexpressing *OsDhn1* shows increased stress resilience [108].
Marker-Assisted Selection (MAS)	Improves stress-resilient rice varieties by incorporating genes related to submergence and salinity tolerance.	*SUB1A* for submergence tolerance and *SalTol* for salinity tolerance integrated into MAS [85,99].
CRISPR/Cas9 Gene Editing	Allows for targeted gene modifications for oxidative stress tolerance; a precision tool for enhancing rice resilience to stress factors.	CRISPR/Cas9 edits stress-responsive genes for improved tolerance [108].
Transcriptomics	Reveals expression profiles of genes involved in salinity and oxidative stress tolerance, aiding genomic breeding efforts.	Identified *Rbohs* and brassinosteroid biosynthesis genes are important for stress tolerance [45].
Proteomics and Metabolomics	Identifies key enzymes and antioxidants involved in oxidative stress management, providing biochemical markers for breeding.	NADP-malic enzyme, glutathione, and ascorbate are critical for oxidative stress control [11].
Abscisic Acid (ABA) Treatment	Enhances stress tolerance by activating antioxidant enzyme activities and maintaining cellular homeostasis under stress conditions.	ABA treatments boost antioxidant enzyme activity for improved stress resilience [11].
Multi-Omics Integration	Provides comprehensive insights into stress-related genes, proteins, and metabolic pathways for a holistic understanding of stress tolerance.	Master regulators (e.g., transcription factors) identified for genetic manipulation [101].
